# Proteotypic Differences of Follicular-Patterned Thyroid Neoplasms

**DOI:** 10.3389/fendo.2022.854611

**Published:** 2022-07-06

**Authors:** Dongdong Huang, Huifang Zhang, Lu Li, Weigang Ge, Wei Liu, Zhen Dong, Jinlong Gao, Nan Yao, Wenxin Fu, Lingling Huang, Tiannan Guo, Yaoting Sun, Xiaodong Teng

**Affiliations:** ^1^ The First Affiliated Hospital, Zhejiang University School of Medicine, Hangzhou, China; ^2^ Westlake Laboratory of Life Sciences and Biomedicine, Key Laboratory of Structural Biology of Zhejiang Province, School of Life Sciences, Westlake University, Hangzhou, China; ^3^ Institute of Basic Medical Sciences, Westlake Institute for Advanced Study, Hangzhou, China; ^4^ Research Center for Industries of the Future, Westlake University, Hangzhou, China; ^5^ College of Pharmaceutical Sciences, Zhejiang University, Hangzhou, China; ^6^ Westlake Omics (Hangzhou) Biotechnology Co., Ltd., Hangzhou, China

**Keywords:** follicular thyroid adenoma, follicular thyroid carcinoma, follicular variant papillary thyroid carcinoma, data-independent acquisition, mass spectrometry

## Abstract

The diagnosis of follicular-patterned thyroid tumors such as follicular thyroid adenoma (FA), follicular thyroid carcinoma (FTC), and follicular variant of papillary thyroid carcinoma (FvPTC) remains challenging. This study aimed to explore the molecular differences among these three thyroid tumors by proteomic analysis. A pressure cycling technology (PCT)-data-independent acquisition (DIA) mass spectrometry workflow was employed to investigate protein alterations in 52 formalin-fixed paraffin-embedded (FFPE) specimens: 18 FA, 15 FTC, and 19 FvPTC specimens. Immunohistochemical (IHC) analysis of 101 FA, 67 FTC, and 65 FvPTC specimens and parallel reaction monitoring (PRM) analysis of 20 FA, 20 FTC, and 20 FvPTC specimens were performed to validate protein biomarkers. A total of 4107 proteins were quantified from 52 specimens. Pairwise comparisons identified 287 differentially regulated proteins between FTC and FA, and 303 between FvPTC and FA and 88 proteins were co-dysregulated in the two comparisons. However, only 23 discriminatory proteins between FTC and FvPTC were detected. Additionally, the quantitative results for ANXA1 expression based on IHC staining and PRM-MS quantification were consistent with the proteomic results, showing that ANXA1 can be used to distinguish FvPTC from FA and FTC. The differentially regulated proteins found in this study can differentiate FA from FvPTC. In addition, ANXA1 is a promising biomarker for differentiating FvPTC from the other thyroid tumors.

## Introduction

Thyroid nodules, encompassing adenomatous nodules, nodular goiters, follicular thyroid adenoma (FA), follicular thyroid carcinoma (FTC), and follicular variant of papillary thyroid carcinoma (FvPTC), are a common finding in adults and exhibit follicular morphological characteristics (1). Given that ultrasound-guided fine-needle aspiration (FNA) biopsy is less traumatic than open surgical resection, it is still the gold standard screening method to validate the characteristics of tumors (2). However, the diagnosis and identification of follicular-patterned thyroid nodules such as FA, FTC, and FvPTC have always been formidable challenges to cytopathologists because of overlapping cytological features and the lack of evidence of capsular or vascular invasion.

Given these difficulties in diagnosing follicular-patterned thyroid neoplasms, ancillary tools are necessary and helpful. Proteomics is a promising approach for identifying biological systems and functions by quantifying and validating large numbers of proteins. This approach has enabled the evaluation and acquisition of some target molecular markers and specific signaling pathways in thyroid pathology. For instance, aiming to identify biomarker candidates to distinguish FA from FTC, Lai et al. discovered several biomarker candidates by a comprehensive mass spectrometry-based analysis; these candidate biomarkers included SUCLG2, with a sensitivity of 75% and a specificity of 80% (3). However, the specific proteomic variants of other follicular-patterned thyroid tumors, such as FvPTC, have not been validated.

FvPTC has been deemed to be entirely or almost entirely composed of follicles, with cells showing nuclear characteristics of classical papillary thyroid carcinoma (cPTC), including intranuclear pseudoinclusions, nuclear grooves, and overlapping nuclei (4). As early as 1960, Lindsay proposed that FvPTC is a distinct subtype of FTC, sharing some biological behaviors with cPTC. However, it was still classified as cPTC during the 1980s, despite the dominance of the follicular pattern (5). A previous study advocated that encapsulated FvPTC behaved like FA or FTC and carried no risk of recurrence or death. In contrast, infiltrative FvPTC was regarded as similar to infiltrative cPTC in terms of biological behaviors and morphological features (6). In addition, the Cancer Genome Atlas research network revealed that the FvPTC group of neoplasms, both infiltrative and noninfiltrative, had the molecular signature of *RAS* mutations, while cPTC exhibits a high prevalence of *BRAF* ^V600E^ mutation (7). There is still debate as to which pathological type FvPTC is closer to. Therefore, research on new potential markers for thyroid pathology, particularly markers enabling FA, FTC, and FvPTC to be distinguished, remains worth conducting.

Recently, pressure cycling technology (PCT) has been developed for semiautomatic processes with small-volume clinical tissues. PCT-data-independent acquisition (DIA) results in higher quantitative accuracy, provides deeper proteome coverage, and is less time-consuming than conventional approaches (8). In this work, we performed the method mentioned above to compare FA, FTC, and FvPTC, gleaning enhanced insights into similarities and differences in protein levels in these three thyroid tumors.

## Methods

### Thyroid Tissue Specimens

As shown in **Table 1**, 345 formalin-fixed paraffin-embedded (FFPE) specimens, specifically, 139 FA, 102 FTC, and 104 FvPTC specimens were obtained from the First Affiliated Hospital of the College of Medicine, Zhejiang University, with approval from the hospital ethics committee. Among the specimens, 18 FA, 15 FTC, and 19 FvPTC specimens were analyzed by the PCT-DIA method to investigate the protein alterations in these three thyroid tumors. These 52 specimens were a subset of our previously analyzed dataset (9). Additionally, immunohistochemical (IHC) analysis of 101 FA, 67 FTC, and 65 FvPTC samples and parallel reaction monitoring (PRM) analysis of 20 FA, 20 FTC, and 20 FvPTC specimens were performed to validate the selected proteins. Two pathologists (X.T. and H.Z.) independently confirmed the pathological diagnosis in the above tissues in accordance with the World Health Organization Classification of Tumors of Endocrine Organs.

**Table 1:** Clinical characteristics in DIA-MS, PRM-MS, and IHC analyses.

	Discovery set	Validation set	
	DIA-MS	PRM-MS	IHC
**Histopathology diagnosis**			
FA	18	20	101
FTC	15	20	67
FvPTC	19	20	65
**Gender**			
Female (%)	35 (67.3%)	41 (68.3%)	160 (68.7%)
Male (%)	17 (32.7%)	19 (31.7%)	73 (31.3%)
**Age at diagnosis**			
Mean	46.77	44.13	46.36
Range	33.24 - 60.3	30.13 – 58.13	32.61 – 60.11
<55 y (%)	34 (65.4%)	44 (73.3%)	157 (67.4%)
≥55 y (%)	18 (34.6%)	16 (26.7%)	76 (32.6%)
**Nodule size**			
Mean	2.76	2.71	2.66
Range	1.13 – 4.39	1.29 – 4.12	1.11 – 4.20
<1 cm (%)	7 (13.4%)	7 (11.7%)	33 (14.2%)
1 - 4 cm (%)	34 (65.4%)	41 (68.3%)	168 (72.1%)
>4 cm (%)	11 (21.2%)	12 (20.0%)	32 (13.7%)

### Proteomic Analysis

Samples (0.6-1.2 mg) were punched from FFPE blocks according to the histopathological areas of interest marked by pathologists (D.H. and H.Z.). FFPE sample preparation was performed using the FFPE-PCT-DIA workflow, as described previously (10, 11). Briefly, FFPE samples were dewaxed and hydrated with heptane and an ethanol gradient (100%, 90%, and 75%). Next, samples were processed by incubation in 0.1% formic acid at 30°C for 30 min and Tris-HCl solution (pH=10, 100 mM) at 95°C for 30 min for decrosslinking. Tissues were subjected to a PCT lysis protocol in 6 M urea and 2 M thiourea buffer. Reduction and alkylation were conducted in 10 mM tris(2-carboxyethyl) phosphine (TCEP) and 40 mM iodoacetamide (IAA). Extracted proteins were digested using LysC (enzyme-to-substrate ratio, 1:40; Hualishi Scientific, China) and trypsin (enzyme-to-substrate ratio, 1:50; Hualishi Scientific, China) by PCT. Digested peptides were desalted on C18 columns (The Nest Group, United States). The chemical reagents described above were purchased from Sigma–Aldrich.

Cleaned peptides (0.4 µg) from each sample were separated on an in-house-developed analytical column (75 µm × 150 mm, 1.9 µm, 100 Å C18 particles) in an Ultimate 3000 HPLC nanoflow system over 45 min in a linear gradient concentration of 3-25% buffer B (buffer A: 2% acetonitrile and 0.1% formic acid in HPLC-grade water; buffer B: 98% acetonitrile and 0.1% formic acid in HPLC-grade water). Eluted peptides were quantified in an Orbitrap (Thermo Q Exactive™ HF) with resolutions of 60,000 and 30,000 (at *m/z* 200 Th) for full MS scans and MS/MS scans, respectively, in DIA mode. For MS/MS scans, we used a series of 24 variable DIA windows to cover the precursor mass over an *m/z* range of 400 to 1200 Th. Each sample was acquired and analyzed by MS with two technical replicates. Therefore, we collected 104 raw DIA data files, which were part of our previous released dataset (9). All DIA data were re-searched by Spectronaut™ (version 13.5) against a thyroid-specific spectral library (12), including 157,548 peptide precursors, 121,960 peptides, and 9941 proteins with a false discovery rate of 0.01. The other parameters were set to the default values.

### Construction of Tissue Microarrays and Immunohistochemical Analysis

Tissue microarray (TMA) blocks for further validation were constructed using samples from the First Affiliated Hospital of College of Medicine, Zhejiang University. Cores (1-mm) were punched from each of the collected specific paraffin-embedded tissue blocks from 101 cases of FA, 67 cases of FTC, and 65 cases of FvPTC and were embedded into new TMAs for subsequent IHC staining. For the current study, some prominent IHC markers, i.e., LUC7L, NUP214, PTK7, DPY30, SYNPO, CDC42EP1, ANXA1, RBM10, FAM50A, MAP2, and CD74, whose differential expression ratio were at least four in each group, were chosen to be verified based on the availability of antibodies for IHC staining. The core from each TMA block was subjected to IHC staining with a specific antibody, verified for accurate specific staining, and scored independently by two pathologists (X.T. and H.Z.). Additionally, a Leica image analysis system was used to measure the staining intensity of each TMA block, which was finally presented as a specific value.

### PRM Quantification and Data Analysis

PRM analysis was performed on 20 FA, 20 FTC (2 specimens were from the same patient), and 20 FvPTC specimens in the Thermo Q Exactive™ HF system connected to a nanoflow DIONEX UltiMate 3000 RSLCnano System. Eighty proteins, including those identified above by IHC staining, whose differential expression ratio was at least eight in each group, were further verified by PRM. Seventy-seven peptide precursors from 65 proteins were successfully programmed for the PRM assay with these criteria applied: unique peptide, no dynamic modification, no missed cleavage, appropriate sequence length, and clear mass-fragment spectrum. Peptides were separated at 300 nL/min along a 60 min 10%–30% linear LC gradient of buffer B. The PRM acquisition method was applied following our previous publication (12).

### Bioinformatical and Statistical Analysis

Statistical diagrams for proteomic data analysis were generated by R software (4.0.2). The coefficients of variation (CVs) were calculated as the ratio of the standard deviation to the mean. Two-tailed paired Student’s *t* test was used to determine probability and was adjusted by the Benjamini and Hochberg (BH) method for generation of volcano plots. The *P* values of the three groups' comparisons were calculated by one-way analysis of variance (ANOVA). The heatmap was generated with the pheatmap R package, and the proteins in each row were subjected to unsupervised clustering. Pathway enrichment analysis was conducted with Ingenuity Pathway Analysis (IPA) software. The protein network was constructed based on the STRING database and plotted with Cytoscape (version 3.8.2). The network edges indicate the interactions with high confidence, with a required minimum interaction score of 0.700. The disconnected nodes in the network were hidden. The Mann–Whitney U test was used to evaluate statistically significant differences in the staining intensity between FA, FTC, and FvPTC specimens for each chosen marker. SPSS version 22 (IBM Corp., Armonk, NY, USA) was used for statistical analyses, and the statistical significance level was set to 0.05.

## Results

### Patient Characteristics and Study Design

**Figure 1:**
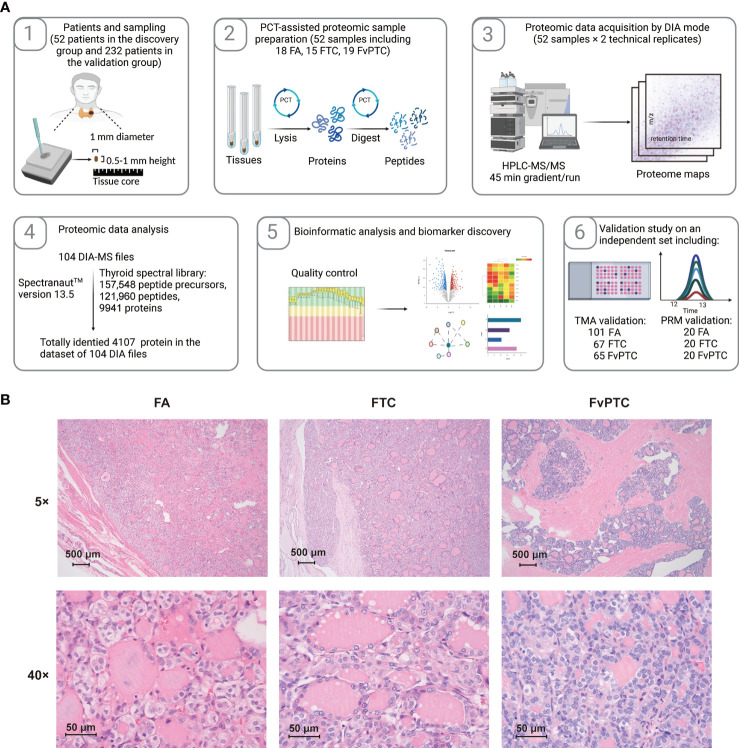
Schematic of the study. **(A)** Workflow of the current study. **(B)** The histopathological characteristics of FA, FTC, and FvPTC.

In the present study, we established two sets of subjects: one contained 52 specimens from 51 patients for proteomic analysis, and the other contained 293 samples from 246 patients for validation by IHC (n=233) and PRM (n=60) analyses. Samples for proteomic analysis were processed *via* a PCT-DIA workflow as described in the *Methods* section (**Figure 1A**).

More details of the clinical characteristics are summarized in **Table 1** and **Table S1**. The histopathological characteristics are shown in **Figure 1B**. FAs are well-encapsulated thyroid nodules without typical invasiveness and abnormal nuclear features, whereas FTCs frequently exhibit capsular disruption, vascular invasion, extrathyroidal extension, and even distant metastasis. In addition, although glandular nuclei, intranuclear inclusions, and nuclear grooves are visible, the histological pattern of infiltrative FvPTC tends to be follicular rather than papillary.

### Proteomic Data Analysis Based on Discovery Group

Two technical replicates of each sample in the discovery set were analyzed for further robustness evaluation of the proteome maps generated from the FFPE tissues. In summary, 52 specimens were analyzed by MS with two technical replicates, and 104 DIA files were subsequently obtained. We identified 4107 proteins based on the 104 proteomic data files. The numbers of identified peptides and proteins in each MS file are shown in **Figures S1A, B**.

**Figure 2:**
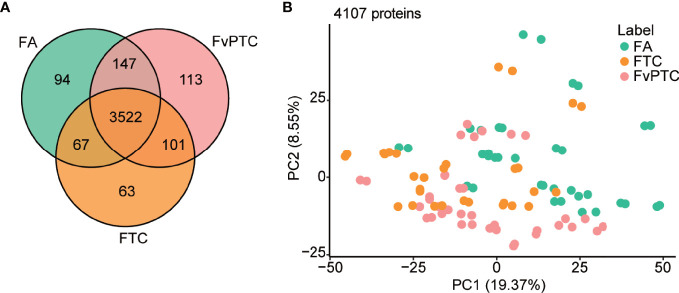
Global proteomic analysis. **(A)** Venn diagram showing protein identification in FA, FTC, and FvPTC. **(B)** Principal component analysis (PCA) using 4107 proteins grouped by tissue type.

For the three groups’ comparisons by one-way ANOVA, *P* values of 799 and 444 proteins are less than 0.05 and 0.01, respectively (**Table S2**). The Venn diagram in **Figure 2A** shows the number of identified proteins displaying significantly quantitative similarities and differences among the three groups. There were 3830, 3753, and 3883 proteins identified in FA, FTC, and FvPTC, respectively. A total of 3522 proteins were shared by all three groups, demonstrating that a large set of overlapping proteins (85.8%) was detected, which validated the robustness of the proteome maps to some extent. In addition, 94 and 63 proteins were only identified in FA and FTC, respectively, while 113 proteins identified in FvPTC were observed in neither FA nor FTC.

In addition, the protein abundance distribution of the two biological replicates of all 52 samples was compared by Pearson correlation analysis (**Figure S1C**), showing the high robustness and reliability of the current comprehensive research. Additionally, principal component analysis (PCA) using 4107 proteins grouped by tissue type (**Figure 2B**) and gender (**Figure S1D**) revealed that FTC is more similar to FvPTC than FA. However, there was no significant difference in gender among these three groups.

### Difference Analysis of Proteomic Profile for the Follicular-Patterned Thyroid Tumors

**Figure 3:**
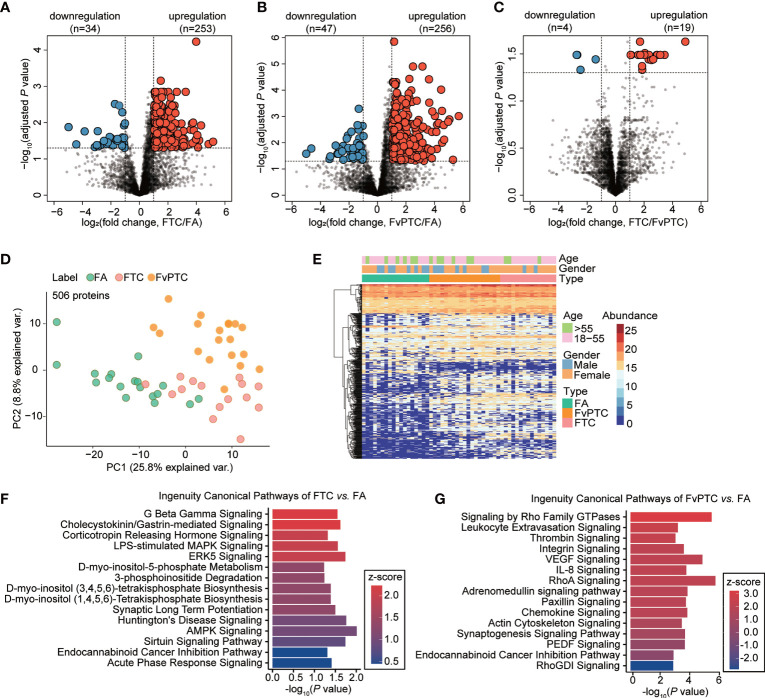
Dysregulated protein expression analysis. **(A-C)** Volcano plot showing dysregulated proteins in **(A)** FTC vs. FA, **(B)** FvPTC vs. FA, and **(C)** FvPTC vs. FTC with a two-fold-change cutoff and an adjusted P value threshold less than 0.05. **(D)** PCA of samples from different tumor types using 506 dysregulated proteins. **(E)** Heatmap showing the protein expression profiles of 52 follicular-patterned thyroid tumor tissue specimens. 506 proteins (rows) are clustered without supervision. Samples (columns) are arranged based on the tissue type. The color indicates the log_2_-transformed intensity of each protein in each sample. Ingenuity canonical pathway analysis was used to enrich pathways from differentially expressed proteins (DEPs) in FTC vs. FA **(F)** and FvPTC vs. FA **(G)**. The top 15 enriched canonical pathways with *P* value <0.05 are ordered based on the z-score values.

Pairwise comparisons of the differential expression of multiple proteins in the FA, FTC, and FvPTC samples were performed to explore apparent similarities and distinctions among the three groups. We first processed proteome profiles showing significantly and differentially altered proteins between FA and FTC. As the volcano plots in **Figure 3A**, by setting a cutoff value of a two-fold change and a threshold adjusted *P* value of less than 0.05, we identified 287 differentially expressed proteins (DEPs), specifically, 253 upregulated and 34 downregulated proteins in FTC. In addition, the comparison showed 303 DEPs in FA and FvPTC, with 256 upregulated proteins and 47 downregulated proteins in FvPTC (**Figure 3B**). Interestingly, only 23 discriminatory proteins were detected between FTC and FvPTC (**Figure 3C**), a much lower number than found in the other comparative analyses. These pairwise analyses of protein expression also showed an apparent separation of FA from FTC and FvPTC, whereas FvPTC showed no apparent distinction from FTC. These results indicated that the two malignant tumors exhibited similar proteotypes but were distinct from the benign tumor FA. Subsequently, we performed PCA (**Figure 3D**) using 506 discriminatory proteins, the combined set of dysregulated proteins from the three volcano plots. These DEPs could distinguish samples from different tumor types better than the complete set of 4107 proteins (**Figure 2B**). In the unsupervised clustering protein heatmap, the protein diversity and abundance were higher in the malignant tumors (FTC and FvPTC) than in FA (**Figure 3E**).

To further investigate the biological function of malignant follicular tumors, we performed pathway and network analyses based on the DEPs. Based on the 287 proteins identified by comparison of FTC and FA (**Figure 3F**), AMPK signaling, which participates in cell growth, autophagy, and metabolism, was the most significantly enriched pathway (13). However, based on the 303 DEPs between FvPTC and FA (**Figure 3G**), RhoA signaling was substantially enriched. Signaling by Rho family GTPases was the most activated pathway, whereas RhoGDI signaling was the most inhibited pathway. In addition, the VEGF signaling pathway was significantly enriched.

### Biological Analysis for the Three Types of Tumors

**Figure 4:**
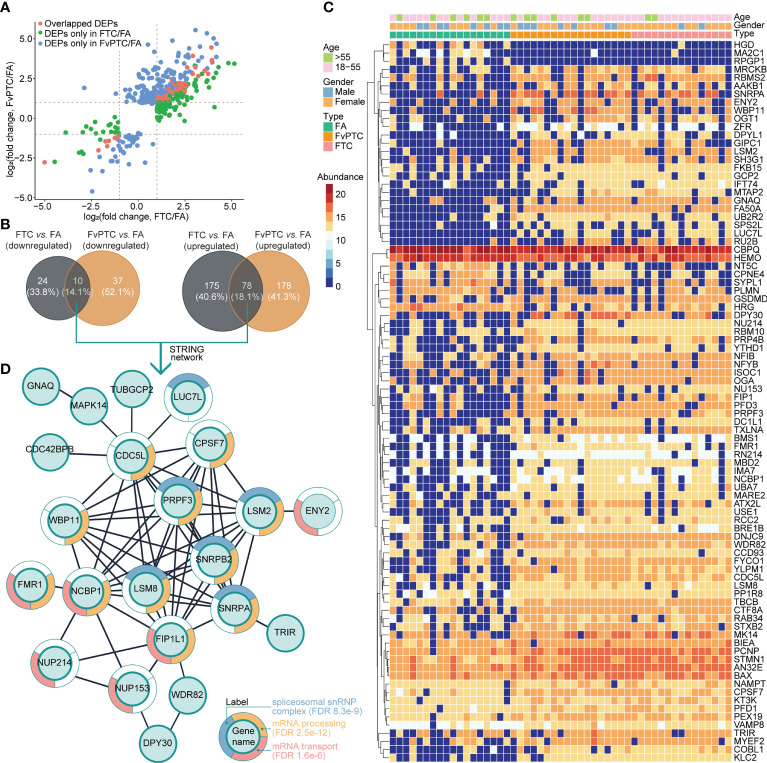
Differentially expressed proteins in malignant tumors vs. the benign tumor and their biological analysis. **(A)** The differentially regulated proteins in FTC vs. FA and FvPTC vs. FA are distributed in the scatter plots. The overlapped DEPs in the two paired comparisons are colored in red, while the unique DEPs in the FTC vs. FA and FvPTC vs. FA are colored in green and blue, respectively. **(B)** Venn diagrams showing the total number of DEPs in FvPTC vs. FA and FTC vs. FA. **(C)** The heatmap exhibited the expression of 88 overlapped dysregulated proteins (panels **A, B**). **(D)** Functional analysis of the largest cluster was obtained by the overlapping upregulated- and downregulated proteins [panels **(A, B)**] using the STRING database. The top-three enriched biological process (BP) terms of gene ontology (GO) after redundancy filtering are visualized on split donut charts around the nodes annotated with those terms.

As indicated above, 253 and 256 proteins were upregulated but 34 and 47 proteins were downregulated in FTC and FvPTC, respectively, compared with FA. The analysis further indicated that FTC and FvPTC shared 78 upregulated and ten downregulated proteins compared with FA (**Figures 4A, B**). These proteins were related to thyroid cancers and their expressions were shown in the heatmap (**Figure 4C**). Of the 88 co-dysregulated proteins, 30 of them were with *P* value less than 0.01 estimated by one-way ANOVA. We further explored the protein-protein interactions of the 88 overlapping proteins by mapping to the STRING database. The largest mapped cluster is shown in **Figure 4D**. The 88 proteins were involved in three major biological functions or processes, namely, spliceosomal snRNP complex (FDR 8.3 e-9), mRNA processing (FDR 2.5 e-12), and mRNA transport (FDR 1.6 e-6), as annotated around the nodes. The key proteins in the center of the network were PRPF3, SNRPB2, SNRPA, LSM2, LSM8, FIP1L1, NCBP1, WBP11, CDC5L, and CPSF7.

### Immunohistochemistry and PRM-MS Quantification for Validation

**Figure 5:**
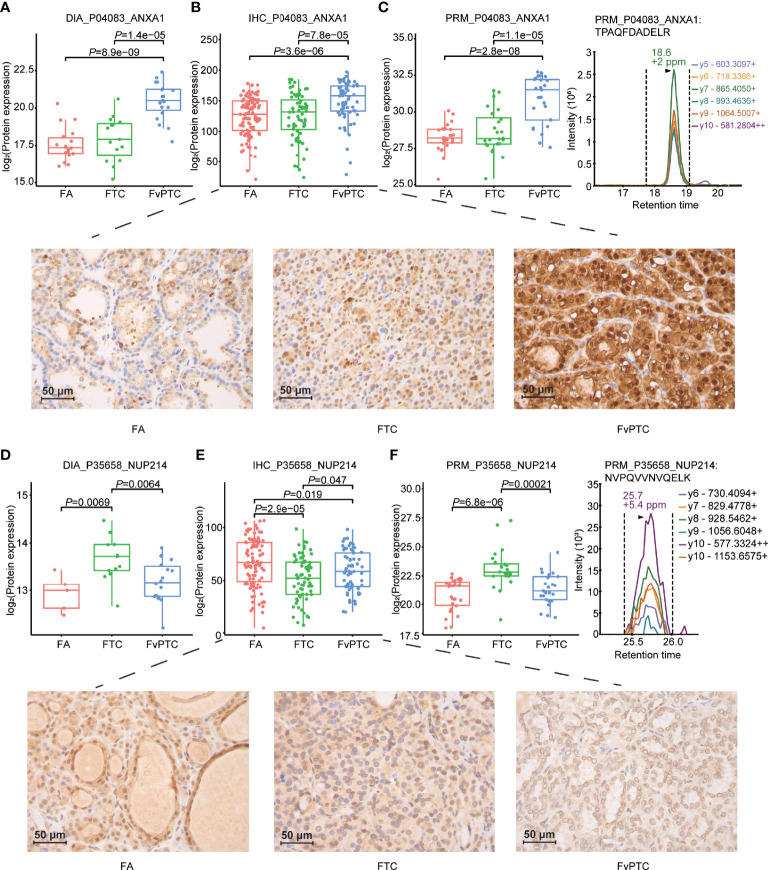
Validation study. Representative results of PCT-DIA, PRM-MS, and immunohistochemical staining of ANXA1 **(A–C)**, NUP214 **(D–F)** in FA, FTC and FvPTC specimens.

Furthermore, we validated the selected proteins as mentioned in the IHC and PRM-MS quantification methods in the two independent sets. Of note, the differential expression levels of ANXA1 in all three tumors were consistent across all three methods including DIA-MS, PRM-MS and IHC. In addition, interestingly, NUP214 presented higher expression as determined by DIA and PRM in FTC compared with FA and FvPTC, although IHC analysis showed different results (**Figure 5**).

## Discussion

Thyroid nodules exhibiting follicular histological features mainly include FA, FTC, and FvPTC. Since there is a wide range of benign to malignant differentiated subtypes, there is no doubt that diagnosing a relatively solitary nodule with follicular morphological characteristics, such as FA, FTC, or FvPTC, is always an enormous challenge for pathologists.

As mentioned above, various studies have recently developed additional methods to provide useful information for diagnosing and treating follicular neoplasms. Based on matrix-assisted Laser Desorption/Ionization (MALDI) Mass Spectrometry Imaging (MSI), a complementary tool with a combination of mass spectrometric data and histology, Yasemin et al. differentiated noninvasive follicular thyroid neoplasms with papillary-like nuclear features (NIFTP) from normal thyroid parenchyma (14). They also revealed that the peptide profiles of NIFTP and encapsulated and infiltrative FV-PTC were similar. In the current work, we utilized PCT-DIA-MS (11) to compare proteomic similarities and differences between FA, FTC, and FvPTC.

Concerning FvPTC, a previous study revealed that encapsulated FvPTC behaves like FA (15). However, microarray expression profiling validated a series of candidate genes involved in molecular mechanisms that differentiate FvPTC from FA (16). Regarding ultrasound characteristics, Ng *et al.* believed that FvPTC and FTC were more likely to be well-defined and noncalcified isoechoic lumps with relatively regular margins (17). In our analysis, 287 and 303 differentially regulated proteins were identified in FTC and FvPTC, respectively, compared to FA. In comparison, only 23 discriminatory proteins were found between FTC and FvPTC, which indicated that these neoplasms share significant similarity with each other and are apparently distinct from FA.

In the comparison of FTC and FA, pathway analysis demonstrated several prominent alterations, such as activated AMPK signaling in FTC. This finding was consistent with a previous study revealing that activation of the AMPK and mTOR pathways can exist simultaneously in FTC (18).

Regarding the biological differences in FvPTC in comparison with FA, our analysis found that RhoA signaling, which has previously been reported to be involved in the proliferation and carcinogenesis of thyroid cancer modulated by miR-128, miR-154-3p, and miR-487-3p, was substantially enriched (19, 20). Interestingly, VEGF signaling was also significantly enriched. Joaquim et al. expounded the role of VEGF signaling in thyroid carcinomas of follicular origin (21). They showed that coexpression of VEGF and two high-affinity tyrosine kinase receptors in VEGF signaling, namely, VEGFR-1/Flt-1 and VEGFR-2/KDR, were expressed in papillary thyroid carcinomas (PTCs), including follicular variants. Notably, VEGFR2 was critical for maintaining thyroid vascular integrity, and its blockade triggered inhibition of vascular remodeling and follicular hypertrophy (22).

Interestingly, the roles of top-ranked discriminatory proteins such as SNRPB2, PRPF3, and LSM8 in other diseases have been investigated for years, although there is limited evidence about the associations between these molecules and malignant thyroid tumors. For example, high expression levels of PRPF3 and SNRPB2 were observed in hepatocellular carcinoma and related to poor prognosis (23, 24). Additionally, LSM8 was significantly correlated with the development of Hashimoto’s thyroiditis, which commonly triggers thyroid cancer (25). Consequently, we hypothesize that the abovementioned costimulatory molecules might trigger oncogenic effects in malignant thyroid tumors.

Besides, in addition to being the most prominent candidate according to the IHC staining and PRM-MS quantification results, ANXA1 has previously been reported in thyroid tumors (26). Its identification in serum and saliva combined with clinical biopsy may distinguish different thyroid nodules, including FTC and cPTC. Regarding NUP214, although there have been few studies on its relationship with thyroid tumors, chromosomal translocations involving the NUP214 locus were found to be recurrent in acute leukemia. Specifically, the C-terminal region of NUP214 is frequently fused with SET and DEK, and these two chromatin remodeling proteins are related to the regulation of transcription (27), which may be associated with the tumorigenesis and progression of FvPTC.

### Limitations

In the current work, although some potential markers for follicular-patterned thyroid tumors were discovered, there are still several limitations. First, at present, it is difficult to differentiate the follicular-pattern thyroid tumors no matter at the pre- and post-surgery. Considering that histopathology is the gold standard for diagnosis, we explored the difference in the FFPE samples instead of FNA samples, which have a clear diagnosis by the experienced pathologist. Without a doubt, the current study is our first step to finding the protein marker candidates for distinguishing the follicular-pattern thyroid tumors. In the next step, we would verify the biomarker candidates in a larger cohort of samples from multiple clinical centers including FNA samples. Second, other follicular-patterned thyroid neoplasms, i.e., Hürthle cell tumors, NIFTP and well-differentiated tumors of uncertain malignant potential, and thyroid nodules with different backgrounds, i.e., hashimoto’s thyroiditis and goiter were not analyzed, which will be analyzed in the further study. Third, the current study lacked comprehensive and integrated analyses combining multi-omic platforms with morphological characteristics based on histopathology or medical ultrasonics, which could be further applied to improve the accuracy and efficacy of diagnostic and prognostic.

## Conclusion

In conclusion, the current proteomic analysis of FA, FTC, and FvPTC identified certain protein signatures that can distinguish various thyroid nodules with different follicular morphological characteristics. Specifically, clusters of proteins demonstrated a marked ability to differentiate FA from FTC and FvPTC. However, more research needs to be carried out to further cull and validate potential biomarker candidates. These findings may provide deeper insight for improving the diagnostic accuracy and efficiency of follicular-patterned thyroid neoplasms.

## Data Availability Statement

The mass spectrometry proteomics data have been deposited to the iProX with the dataset identifier IPX0003973000.

## Ethics Statement

The studies involving human participants were reviewed and approved by Clinical Research Ethics Committee, The First Affiliated Hospital, Zhejiang University School of Medicine. Written informed consent for participation was not required for this study in accordance with the national legislation and the institutional requirements.

## Author Contributions

XT, DH, and YS designed the project. XT, DH, and HZ revised the slides and collected the FFPE samples. YS, LL, and WL performed the experiments for discovery set. NY and WF performed the targeted proteomic sample preparation and analysis. DH and HZ did the IHC validation study. YS, WG, and LH conducted proteomic data analysis. DH and YS wrote the manuscript with inputs from all co-authors. TG supported the data analysis and data presentation in the manuscript. XT and YS supervised the project. All authors contributed to the article and approved the submitted version.

## Funding

This work is supported by grants from the National Key R&D Program of China (No. 2021YFA1301601, 2021YFA1301602, 2020YFE0202200), Zhejiang Provincial Research Center for Cancer Intelligent Diagnosis and Molecular Technology (JBZX-202003), China Postdoctoral Science Foundation (2021TQ0283) and International Postdoctoral Exchange Fellowship Program (Talent-Introduction Program) (YJ20210170).

## Conflict of Interest

TG is a shareholder of Westlake Omics Inc. WG, YN, WF. and LH are employees of Westlake Omics Inc.

The remaining authors declare that the research was conducted in the absence of any commercial or financial relationships that could be construed as a potential conflict of interest.

## Publisher’s Note

All claims expressed in this article are solely those of the authors and do not necessarily represent those of their affiliated organizations, or those of the publisher, the editors and the reviewers. Any product that may be evaluated in this article, or claim that may be made by its manufacturer, is not guaranteed or endorsed by the publisher.

## Supplementary Material

Supplementary Figure 1 Data quality control analysis. **(A)** Identified peptide and **(B)** protein numbers in each DIA run. **(C)** Pearson correlation analysis of technical replicates for each paired sample. **(D)** Principal component analysis (PCA) using 4107 proteins grouped by gender.

Supplementary Figure 2 IHC images of ANXA1 and NUP214 in FA, FvPTC, and FTC specimens.

Supplementary Table 1 Details of the clinical characteristics.

Supplementary Table 2 Protein expression comparison among the FA, FTC, and FvPTC.
