# Supplementary figures and images for: Proteotypic Differences of Follicular-Patterned Thyroid Neoplasms
Source: Front Endocrinol (Lausanne). 2022 Jul 6;13:854611. doi: 10.3389/fendo.2022.854611 (PMC9340356; doi:10.3389/fendo.2022.854611)

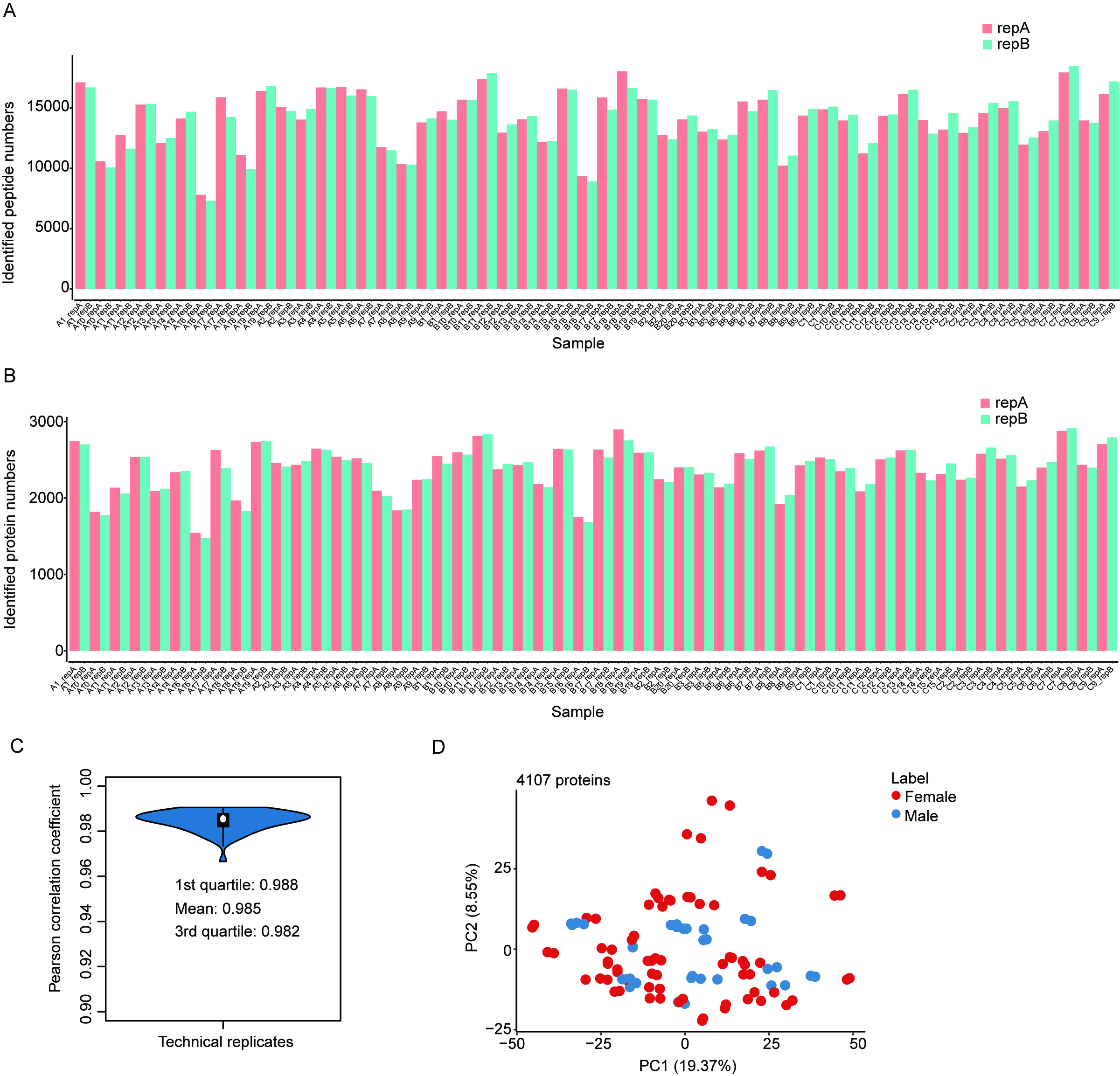

Supplement: Supplementary Figure 1 — Data quality control analysis. (A) Identified peptide and (B) protein numbers in each DIA run. (C) Pearson correlation analysis of technical replicates for each paired sample. (D) Principal component analysis (PCA) using 4107 proteins grouped by gender. [file Image_1.tif]

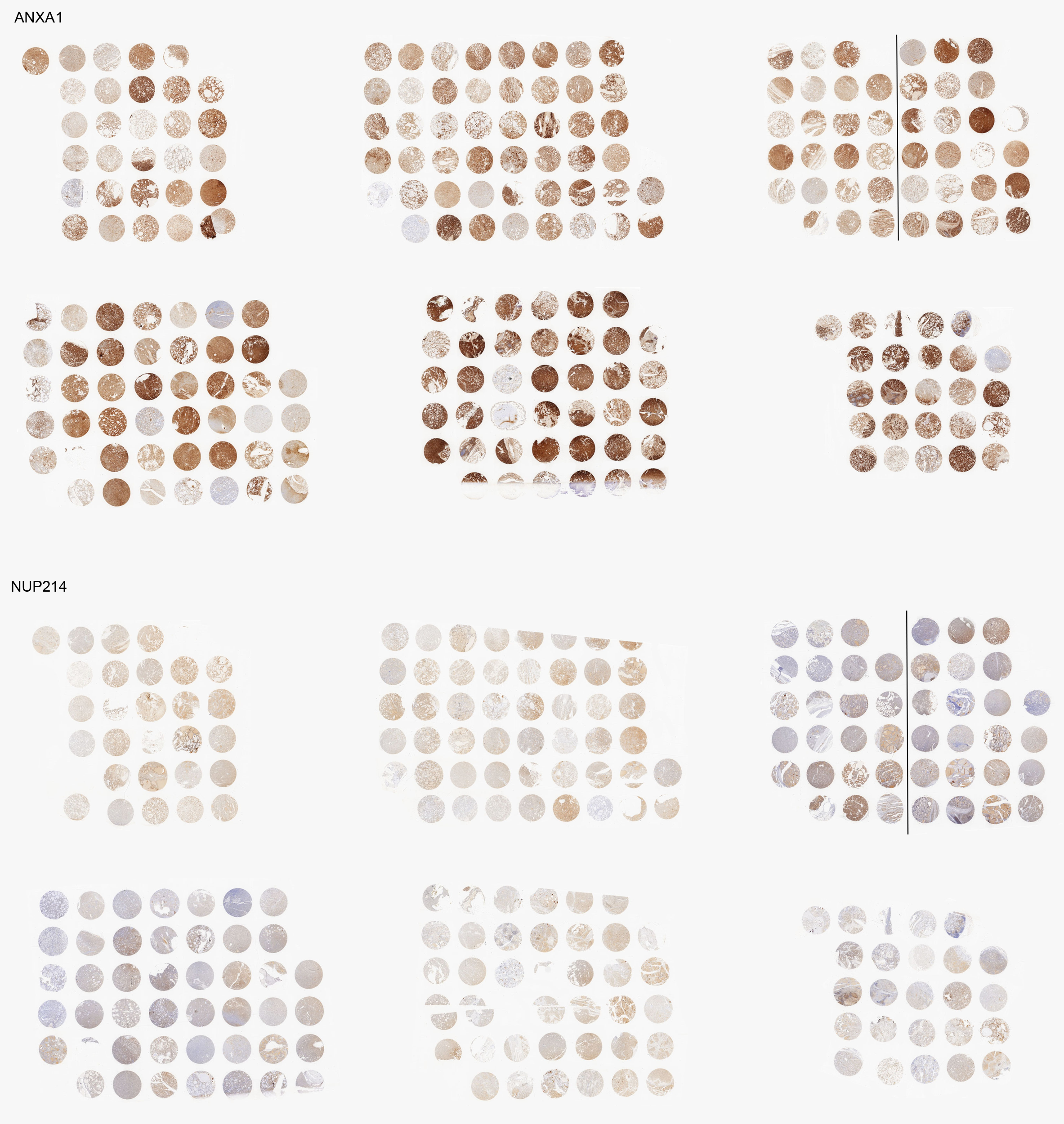

Supplement: Supplementary Figure 2 — IHC images of ANXA1 and NUP214 in FA, FvPTC, and FTC specimens. [file Image_2.tif]
